# Effectiveness and safety of hypotensive resuscitation in traumatic hemorrhagic shock: A protocol for meta-analysis

**DOI:** 10.1097/MD.0000000000018145

**Published:** 2019-11-27

**Authors:** Hua Wang, Mao-Bing Chen, Xu-Wen Zheng, Qi-Han Zheng

**Affiliations:** a Department of ICU, Wujin People Hospital; b Department of Emergency, Wujin People Hospital Affiliated with Jiangsu University, and the Wujin Clinical College of Xuzhou Medical University, Changzhou Jiangsu, PR China.

**Keywords:** hemorrhagic, hypotensive resuscitation, meta-analysis, resuscitation, shock, shock, traumatic

## Abstract

**Background:**

Hypotensive resuscitation is an old study. But its benefits and losses are still controversial. In clinic, the method of fluid resuscitation needs more reliable experimental evidence. This study's objective is to systematically evaluate the efficacy of hypotensive resuscitation in patients with traumatic hemorrhagic shock.

**Methods and analysis:**

Through October 2019, Web of Science, PubMed, the Cochrane Library, EMBASE, and Clinical Trials will be systematically searched to identify randomized controlled trials exploring the efficacy of hypotensive resuscitation in traumatic hemorrhagic shock. Strict screening and quality evaluation will be independently performed on the obtained literature by 2 researchers; outcome indexes will be extracted, and a meta-analysis will be performed on the data using Revman 5.3 software.

**Ethics and dissemination:**

The stronger evidence about the efficacy of hypotensive resuscitation in traumatic hemorrhagic shock will be provided for clinicians.

**Trial registration number:**

PROSPERO CRD42019133169.

**Strengths of this study:**

This study is not only a simple combination of data, but also to verify and discuss the reliability of the results, and provide more convincing evidence for clinicians.

**Limitations of this study:**

Firstly, according to the previous literature researching, it is found that the number of relevant randomized controlled trials is small and the quality level of the literature is uneven. Secondly, the efficacy of hypotensive resuscitation is discussed for a long time, different trials may take place at different times. Comparability between different trials is reduced.

## 1 Introduction

With the increase of vehicles and large machinery, trauma has become the most common cause of death for the people under 44 years old,^[1]^ with more than 5 million patients dying from trauma globally every year.^[2]^ Blood loss is one of the main cause of death from trauma. Once hemorrhagic shock occurs, the mortality of patients will greatly increase.^[3]^ Traumatic hemorrhagic shock refers to a series of reaction syndromes caused by injury to and bleeding of the viscera, decreased effective circulating blood volume and insufficient microcirculation perfusion.^[4,5]^

The most effective treatment for hemorrhagic shock is usually surgery, but how resuscitation should be chosen prior to surgery has not been determined.^[6,7]^ At present, there are 2 methods of resuscitation in patients with traumatic hemorrhagic shock: aggressive resuscitation and hypotensive resuscitation.^[8]^ In traditional resuscitation therapy, it is emphasized that the effective circulating blood volume should be restored as soon as possible, which is called aggressive resuscitation.^[9,10]^ Then, the concept of hypotensive resuscitation was proposed. The difference between aggressive resuscitation and hypotensive resuscitation is targeted blood pressure management.^[11]^ Hypotensive resuscitation is better than aggressive resuscitation in theory, but it is lack of RCTs supporting. Is it because the sample size is too small, or the differences between 2 resuscitations are too small? It is unknown which method is preferable in the real world setting. This study attempts to clarify the differences between aggressive resuscitation and hypotensive resuscitation, aiming to provide evidence for the treatment of traumatic hemorrhagic shock.

In the preliminary data preparation process, we found that there was a similar previous meta-analysis study,^[12]^ but we found that their results were not stable, and the evidences ware not reliable. It included too many low-quality studies, and its disposition was flawed. Therefore, this meta-analysis is conducted to obtain a more stable and convincing conclusion through various methods.

## 2 Methods

### 2.1 Design and registration

A meta-analysis will be conducted to systematically evaluate the efficacy of hypotensive resuscitation in patients with traumatic hemorrhagic shock. This protocol has been registered on the international prospective register of systematic reviews (PROSPERO), registration number: CRD42019133169 (https://www.crd.york.ac.uk/PROSPERO). No ethical approval is required since this study used data that were already in the public domain.

### 2.2 Study selection

#### 2.2.1 Study type

This study will include Randomized Controlled Trials (RCTs).

#### 2.2.2 Study object

The study objects had the following requirements:

1. adult patients;
2. a clear diagnosis of shock; and
3. trauma and blood loss.

#### 2.2.3 Intervening measure

Patients will be randomized into 2 groups: the hypotensive resuscitation (HR) group and the conventional resuscitation (CR) group. In the hypotensive resuscitation group, patients will receive less fluid resuscitation, keeping their blood pressure at a lower level, while in the conventional resuscitation group, patients will receive conventional fluid resuscitation, keeping their blood pressure at a normal level.

#### 2.2.4 Outcome indicator

The following outcomes will be compared between the HR group and CR group:

1. overall mortality,
2. 24-hour mortality.

#### 2.2.5 Exclusion criteria

Studies that investigated other causes of shock including neurogenic shock, surgical bleeding shock, and hysterorrhexis shock will be excluded. Studies with data that could not be extracted or utilized, animal experiments, and literature reviews, and other types of non-experimental articles will be excluded.

### 2.3 Data sources and searches

**Figure 1:**
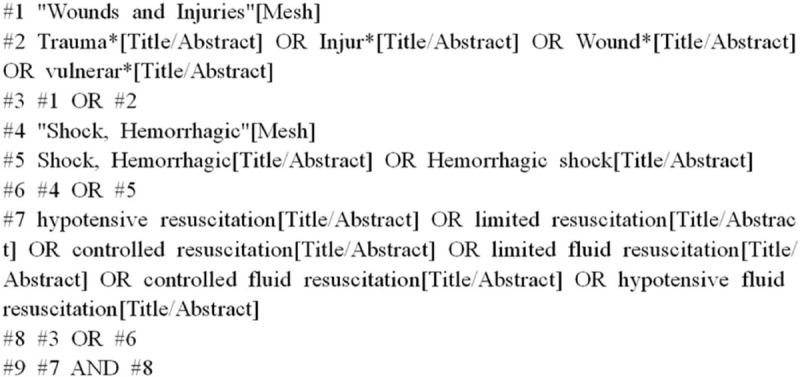
Retrieval strategy for the PubMed database.

We searched English language publications through September 2019 using the following databases: Web of Science, PubMed, the Cochrane Library, EMBASE, and Clinical Trials. The search terms were “hypotensive resuscitation”, “hemorrhagic shock”, and “trauma”. Here, we use the PubMed database as an example (Fig. 1).

### 2.4 Study screening, data extraction, and risk assessment of bias

Data will be collected independently by 2 researchers. The unqualified studies will be eliminated, and the qualified ones will be screened by reading the title, abstract, and full text. Then, the research data will be extracted and checked, and disagreements will be discussed or a decision was made by the author. The extracted data will include the following:

1. basic information of the study, including title, author, and year of publication;
2. characteristics of the included study, consisting of study duration, sample sizes of the intervention group and the control group, and intervention measures;
3. outcome indicators and the data included; and
4. the elements needed to perform a risk assessment for bias.

The risk of bias in the included studies will be assessed using the RCT bias risk assessment tool recommended in the Cochrane Handbook for Systematic Reviews of Interventions (5.1.0).

### 2.5 Statistical analysis

Revman 5.3 software will be used for the meta-analysis. The relative risk (RR) will be used as an effect indicator for dichotomous variables, and the estimated value and 95% confidence interval (CI) will be included as effect analysis statistics. A heterogeneity test will be conducted with the results of each study. To make the results more stable, the random effects model, a more conservative effects model, will be adopted in this meta-analysis. The significance level will be set at α = 0.05.

### 2.6 Subgroup analysis

Subgroups will be established based on quality evaluation of studies.

### 2.7 Assessment of publication bias

If more than 10 articles are available for quantitative analysis, we will generate funnel plots to assess publication bias. A symmetrical distribution of funnel plot data indicates that there is no publication bias, otherwise, we will analyze the possible cause and give reasonable interpretation for asymmetric funnel plots.

### 2.8 Confidence in cumulative evidence

GRADE^[13]^ system will be used for assessing the quality of our evidence. According to the grading system, the level of evidence will be rated high, moderate, low, and very low.

## 3 Discussion

Hypotensive resuscitation is an established method.^[14]^ Although guidelines and expert consensus recommend HR for resuscitation in patients with traumatic hemorrhagic shock, there are few RCTs to support this recommendation.^[15,16]^ The absence of evidence from high-level evidence-based medicine is a source of concern. The purpose of this meta-analysis was not to draw a positive or negative conclusion but, more importantly, to analyze the stability of the conclusion.

First, we found that the CNKI database was retrieved in the previous meta-analysis. I take a negative attitude towards this. Including the CNKI database could increase the number of studies included, but after carefully reading the full texts of these available studies, it was determined that the quality of research in the CNKI database is uneven. After weighing quantity and quality, this meta-analysis will not include the CNKI database. Then compared with the previous meta-analysis, the RCT bias risk assessment tool recommended in the Cochrane Handbook for Systematic Reviews of Interventions will be used. By analyzing the random sequence generation, allocation concealment, blinding method, incomplete outcome data, selective reporting, etc, to evaluate the quality of the research.

Researchers think that during the early stages of trauma, aggressive resuscitation could reduce the patient's core temperature, and excessive infusion could dilute the clotting factor. Meanwhile, raising blood pressure could result in the loss of red blood cells, leading to hypoxia in tissues and acidosis. These 3 conditions (hypothermia, clotting disorders, acidosis) are referred to as the triangle of death.^[17]^ Hypotensive resuscitation early after trauma provides the fluid needed to maintain the basic functions of the body, which can reduce bleeding and improve tissue hypoxia, theoretically reducing mortality and improving the patient's prognosis.^[18]^

Why are the results of RCTs not as good as the theory? In actual clinical practice, it is difficult to control blood pressure in a short time to the target pressure. In hypotension resuscitation, the fluctuation of blood pressure will inevitably cause tissue ischemia and hypoxia, and cause tissue damage. This might be HR's weakness.

This study will conduct a meta-analysis of related RCTs, and provide evidence on the efficacy and safety of hypotensive resuscitation in traumatic hemorrhagic shock treatment, so as to better guide clinical practice.

Hua Wang orcid: 0000-0002-7288-5907.
